# Perspective: Methods matter: highlighting key methodological contributions in nursing research

**DOI:** 10.1177/17449871251370602

**Published:** 2025-09-17

**Authors:** Pernilla Garmy

**Affiliations:** Professor, Kristianstad University, Kristianstad, Sweden

Nursing research rests on the need for robust and reliable methods. To draw well-founded conclusions from empirical studies, research must have a solid scientific foundation.

The *Journal of Research in Nursing* primarily publishes studies that explore various aspects of care and nursing. However, throughout the journal’s impressive 30-year history (it was founded in 1996), several important methodological papers have also been published.

In this Collection – a set of papers with a shared theme – we highlight both older and more recent methodological contributions. What makes methodological papers particularly valuable is their long-term relevance. A method often becomes more robust the more it is used, tested and developed over time.

The most cited paper in the *Journal of Research in Nursing’s* 30-year history is ‘Memoing in qualitative research’ by Professor Melanie Birks and colleagues (2008). At the time of publication, Melanie Birks was still a doctoral student at Monash University in Australia. Today, she is a professor at James Cook University, also in Australia.

The paper addresses the memos that researchers write in the context of qualitative research. In a style that is both personal and methodologically rigorous, Birks et al. described how such notes can serve as memory aids throughout the research process and document decisions made along the way. The authors generously shared their own experiences while also drawing on a solid theoretical foundation.

A central contribution in the paper is the acronym MEMO, which summarises four key principles:

• Mapping research activities
• Extracting meaning from data
• Maintaining momentum
• Opening communication

The first principle, Mapping research activities, involves documenting the research process – akin to diary entries, such as a doctoral student noting decisions made during a supervision meeting.

Extracting meaning from data refers to formulating insights during data coding. The authors described memos as the vehicle that transports the researcher from the concrete to the conceptual.

The third principle, Maintaining momentum, is about staying close to the data and avoiding overly ambitious interpretations. Here, researchers can write down their own perspectives to later identify potential preconceptions or biases. Memo-writing is a flexible and creative process that allows uncertainty. The authors encouraged researchers to take intellectual risks – it is both permitted and often fruitful to revise one’s memos along the way. In this way, memos support the researcher’s reflections in a process that is often non-linear.

The final principle, Opening communication, highlights memos as a tool for dialogue. Although they are often written for personal use – such as between a doctoral student and supervisor – they can also serve as a basis for discussions with colleagues, research teams or other stakeholders. Sharing memos can open up new perspectives and ideas (Birks et al., 2008).

Although Birks et al. (2008) invited us into the reflective and personal aspects of the research process through memo-writing, Campbell et al. (2020) shifted our attention to another crucial decision in qualitative research – the selection of participants. Their paper on purposive sampling further reinforces the importance of methodological clarity from the earliest stages of a study.

Campbell et al. (2020) had written an insightful paper on purposive sampling. The aim is to clarify what purposive sampling is, why it is used, and how it can be applied in qualitative research - particularly in nursing. The paper illustrates this through three case studies where the method has been applied in different ways, adapted to the studies’ respective designs and contexts.

It is clear that purposive sampling contains not only shared principles but also room for variation depending on the research question and context. By linking the sampling to the study’s aims and methodology, it contributes to strengthening the trustworthiness of data collection and analysis.

The paper relates to four central criteria for research quality: credibility, transferability, dependability and confirmability. Campbell et al. particularly emphasised the importance of novice researchers making deliberate sampling choices that support methodological rigour, rather than relying on convenience. The paper also shows how well-considered sampling can help decision-makers better understand the relevance and applicability of research.

If sampling forms the foundation for what data we gather, the overall design provides the framework for how we make sense of it. Doyle et al. (2020) built on this by presenting qualitative descriptive design as an accessible and effective approach, particularly for practice-focused nursing research.

Doyle et al. (2020) provided an overview of qualitative descriptive design in nursing research and demonstrated its relevance in clinical contexts where practical utility is key. The method is characterised by its simplicity, flexibility, and practical applicability; making it especially suitable for studies at master’s level. Here, clinically active nurses who are often new to research get the opportunity to explore pressing questions in their own care environments – questions that may lead to concrete improvements, rather than theoretical expansion.

Doyle et al. noted that qualitative content analysis and thematic analysis are the most used analytical methods within this design. At the same time, the authors pointed out that the method has occasionally been criticised for lacking rigour, particularly in cases of unclear methodological choices or limited transparency. It can also be difficult to distinguish what characterises descriptive design in relation to other qualitative approaches. Through practical examples from healthcare research, the paper shows how the method can be applied throughout the research process and clarifies its underlying philosophical foundations and key characteristics. It thus strengthens our understanding of how qualitative descriptive design can be used in a methodologically sound and trustworthy way in clinically oriented research.

Dhollande et al. (2021) turned our attention to another central activity in the research cycle: reviewing and synthesising existing evidence. Their guidance on integrative reviews is particularly valuable for novice researchers seeking to develop structured and credible literature overviews.

Dhollande et al. (2021) had written a clear methodological paper on how to conduct integrative literature reviews. Integrative reviews – which include both qualitative and quantitative studies – contribute to a comprehensive understanding of a research field but require clear structure and a systematic approach to ensure the reliability of findings.

The paper offers a practical framework for nurses who are new to research and wish to undertake an integrative review. The authors described and exemplified established methods for formulating research questions, conducting literature searches, extracting and critically appraising data and analysing the results. By applying these steps transparently and systematically, the reproducibility of the review is strengthened, along with readers’ confidence in the findings. The paper demonstrates how the use of structured tools can support novice researchers and enhance the quality of integrative reviews in nursing.

Taken together, these methodological contributions form a solid resource for researchers at different stages of their journey – from designing new empirical studies to critically appraising existing knowledge. But beyond their individual strengths, they also remind us of the broader responsibility we carry: to make informed, transparent, and coherent methodological choices.

Reading methodological papers is a fundamental part of research. These are the texts we turn to when we move from planning to conducting and applying research. They provide guidance in the many methodological decisions we inevitably must make as researchers – because we cannot keep all options open indefinitely. Choices must be made. And it is reassuring to be able to lean on a well-written methodological paper and say: ‘Yes, I am aware that other options existed – but I followed the guidance in this reference, and according to it, this is the recommended way to proceed’.

I encourage you as a researcher to engage with several methodological papers – but within a single study, it is often beneficial to clearly anchor your work in one, or a few methodological sources, and follow them consistently. This often leads to a more coherent and methodologically sound manuscript, as opposed to referencing a wide range of, sometimes contradictory, methodological literature. It is difficult – perhaps even impossible – to remain loyal to multiple methodological frameworks within one study, since each approach entails its own choices and priorities.

That said, this does not mean that you, as a researcher, must commit to one single approach for all future projects. On the contrary – methodological diversity is valuable. But each individual study often benefits from a clear methodological direction and internal coherence.

As a next step, I hope to receive more methodological manuscripts for the *Journal of Research in Nursing*. We welcome both major and minor methodological approaches. Have you tried something new? Would you like to describe a specific part of the research process? Let yourself be inspired by Melanie Birks, who was still a doctoral student when she wrote her now widely cited paper on memoing. Who knows – perhaps your methodological paper will be the next one others turn to for guidance.
